# Antibiotic Resistance: From Pig to Meat

**DOI:** 10.3390/antibiotics10101209

**Published:** 2021-10-05

**Authors:** Xavier C. Monger, Alex-An Gilbert, Linda Saucier, Antony T. Vincent

**Affiliations:** 1 Département des Sciences Animales, Faculté des Sciences de l’Agriculture et de l’Alimentation, Université Laval, Quebec City, QC G1V 0A6, Canada; xavier.monger.1@ulaval.ca (X.C.M.); alex-an.gilbert.1@ulaval.ca (A.-A.G.); Linda.Saucier@fsaa.ulaval.ca (L.S.); 2 Institut sur la Nutrition et les Aliments Fonctionnels, Faculté des Sciences de l’Agriculture et de l’Alimentation, Université Laval, Quebec City, QC G1V 0A6, Canada

**Keywords:** animal production, antibiotic resistance, environment, meat, microbiota, one health, pathogens, swine

## Abstract

Pork meat is in high demand worldwide and this is expected to increase. Pork is often raised in intensive conditions, which is conducive to the spread of infectious diseases. Vaccines, antibiotics, and other biosafety measures help mitigate the impact of infectious diseases. However, bacterial strains resistant to antibiotics are more and more frequently found in pig farms, animals, and the environment. It is now recognized that a holistic perspective is needed to sustainably fight antibiotic resistance, and that an integrated One Health approach is essential. With this in mind, this review tackles antibiotic resistance throughout the pork raising process, including their microbiome; many factors of their environment (agricultural workers, farms, rivers, etc.); and an overview of the impact of antibiotic resistance on pork meat, which is the end product available to consumers. Antibiotic resistance, while a natural process, is a public health concern. If we react, and act, collectively, it is expected to be, at least partially, reversible with judicious antibiotic usage and the development of innovative strategies and tools to foster animal health.

## 1. Introduction

With an ever-increasing human population, there is a constant pressure to intensify productivity in many agricultural sectors, including the production of meat as a source of protein. Preventing and treating infections is key to productivity and contributes to the well-being of the farmed animals raised for meat. Depending on the animal density on farms and the biosecurity measures in place, infectious diseases can spread rapidly, infecting one or more herds, and cause high levels of mortality. In the absence of treatment, this can lead to a loss of productivity and profit for the producers [1]. Although raising farm animals without antibiotics has been achieved, some will argue that mortality rates and welfare issues are against animals’ rights to be treated when infections strike. Therefore, antibiotics continue to be used on livestock farms [2].

Unfortunately, extensive use of antibiotics has increased the pressure for the selection of bacterial strains resistant to the various antimicrobial compounds [3]. Antibiotic-resistant bacteria are now frequently isolated from most farmed animals [4]. Moreover, the antibacterial compounds and resistant bacteria can spread outside agricultural regions and accelerate the resistance phenomenon in other bacteria, including those pathogenic to humans [5]. Several other factors, such as increased climate change and reduced biodiversity, will force pathogens to evolve and to adapt rapidly [6]. Although evolution is generally a slow process requiring many generations, bacteria can modulate their gene repertoire within just one generation, and thus radically change their behaviour. Mobile genetic elements (MGEs), such as plasmids, transposons, and integrons, facilitate horizontal movement of genes between species and can promote rapid evolution of bacteria, including the acquisition of virulence factors or resistance to antibiotics [7,8,9]. 

The occurrence of resistance is increasingly threatening the efficacy of antibiotics, including those historically used in human medicine to alleviate the burden and suffering caused by many infections [10]. While discovering penicillin, Alexander Fleming observed organisms capable of resistance and warranted caution in its usage [11] and today antibiotic-resistant and persister cells are studied extensively [12,13]. Time has proven him right, as the World Health Organization has declared that around 700,000 deaths every year can be attributed to diseases that have become difficult to treat because of antibiotic resistance. If the problem continues to increase at the rate it has done so far, the number could reach 10,000,000 by 2050 [14]. It is therefore mandatory to adopt a One Health approach to tackle the biotic and abiotic components that participate in the phenomenon of antibiotic resistance. A better understanding of how antibiotic resistance is evolving throughout the agri-food chain is essential to the effectiveness of the One Health approach. Unfortunately, while 125 countries declare having some antimicrobial resistance awareness activities, only 36 of those are taking measures to counter the problem in the agricultural sector [15]. This suggests that the impacts of agriculture on antimicrobial resistance may be underestimated and requires further attention and investigations.

**Figure 1:**
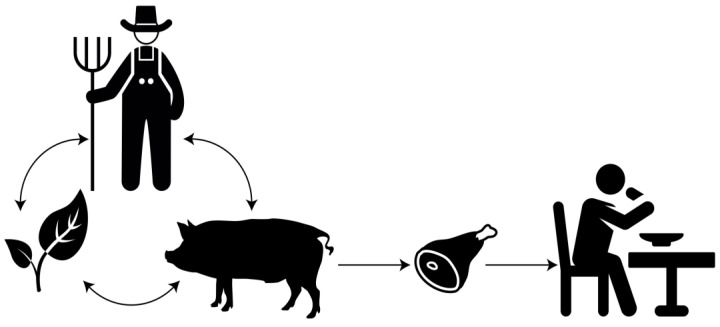
Representation of several interconnected components influencing the quality of pork meat.

Pigs are, without any doubt, among the most important farm animals in both numbers and biomass [16], despite religious restrictions in some cultures. As a matter of fact, the UN’s Food and Agriculture Organization predicts that pigs farming is the animal industry that will experience the strongest growth, with an expected increase of 8.6% by 2030 and 12.7% by 2050 [16]. The swine industry plays a key role in the food supply chain and has a high economic impact. In 2018, pork production reached a value of $94 billion USD worldwide [16]. Despite regulatory differences between countries, the pork industry, like other sectors, uses antibiotics and must try to find a balance between animal welfare, consumer expectations, and sustainable development. It is more necessary than ever before to structure and compile our knowledge of antibiotic resistance and to help producers to sustainably provide good quality pork meat while maintaining a good yield. This review will contribute to a better understanding of antibiotic-resistant bacteria transmission throughout the pork value chain and explore possible solutions (Figure 1).

## 2. From a Pig Perspective

### 2.1. Therapeutic Use of Antibiotics

The use of antibiotics was introduced in animals soon after their discovery by Sir Alexander Fleming in 1929 [17]. One of the first commercially successful antibiotics was Bayer’s Prontosil, a sulfonamide derived from azo dye [18]. As reviewed elsewhere, soon after the production of Prontosil, antibiotics began to be used in agriculture [19]. Initially, antibiotics were seen exclusively as molecules to reduce animal mortality, and not as growth promoters, although this application also quickly emerged.

The human population is growing quickly, and the rate is likely to accelerate. The need for animal protein will increase correspondingly, as will the densification of farmed animals. It is estimated that the pork industry is going to have a marked increase in antibiotic consumption by 2030 [20].

Although antibiotics are banned as growth promoters in several countries, they are still used for the prevention, control, or treatment of infectious diseases, as stated by the American Veterinary Medical Association (AVMA) [21]. Many studies were dedicated to identifying pathogenic bacteria that are problematic for the pig industry and assess their level of risk in terms of antibiotic resistance. One study investigated more than 57,000 published articles over 50 years on 40 pathogens in pigs [22]. The most studied bacterial pathogens worldwide, and therefore probably some of the most problematic for the industry, are *Salmonella, Escherichia coli, Actinobacillus pleuropneumoniae* and *Pasteurella multocida*. Finally, the authors noted an increase in strains of *Streptococcus suis* (*S. suis*), a major porcine pathogen causing septic diseases resistant to penicillin, tetracycline and macrolides [23].

Holmer et al. recently investigated the evolution of antibiotic resistance in Denmark from 2004 to 2017 for the main pathogenic bacteria in pigs and reported that approximately 70% of isolated *E. coli* strains were resistant to tetracycline and streptomycin, and a significant number of strains were also resistant to other antibacterial compounds [24]. There was a marked increase in *E. coli* strains resistant to florfenicol, which correlated with its usage. Interestingly, florfenicol is usually not prescribed for gastrointestinal infections such as those produced by *E. coli*, but for respiratory ones. Holmer et al. have argued that the increase in strains of *E. coli* resistant to florfenicol may be due to co-selection with another given antibiotic or when pigs are treated for respiratory infections. Another study demonstrated that using tetracycline in pigs prompted co-selection for resistance genes for aminoglycosides and tetracycline [25]. These studies illustrate the importance of taking a wider view of antibiotic resistance.

The province of Quebec in Canada is a major producer of pigs and pork meat. According to the latest report from the agriculture, fisheries and food ministry (MAPAQ), many isolates of *E. coli* and *Salmonella* spp. are resistant to more than three classes of antibiotics [26]. While this same report indicates a stable level of resistance for *Salmonella*, and even a decrease in resistance to trimethoprim/sulfamethoxazole, florfenicol, and tetracycline, it reveals a marked increase of resistance in *E. coli* for many antibiotics of the aminoglycoside, β-lactam, phenicol, and quinolone classes.

A recent meta-analysis has shown that tetracycline is one of the most widely used antibiotics worldwide for pigs, and that tetracycline resistance genes are some of the most abundant antibiotic resistance genes (ARGs) observed in the pig microbiome [27]. However, Burow et al.’s, 2019 study demonstrated that the baseline level of resistance to tetracycline is naturally elevated, even in animals raised without this antibiotic [28]. Burow et al. mention that this high basal resistance level could potentially be explained by facilitated co-selection for tetracycline resistance when another antibiotic is used, such as trimethoprim. This is corroborated by another study that showed that contact with animals treated with antibiotics is sufficient to transfer resistance in bacteria of untreated animals [29]. Stanton et al. observed the presence of bacteria resistant to tetracycline, even in pig farms that raised pigs organically for at least 4 years [30]. The organically raised pigs had higher counts of tetracycline resistance bacteria than feral pigs. The presence of bacteria resistant to other classes of antibiotics has been recorded in various organic pig farms in Europe, although to a lesser extent than in conventional farms [31]. These studies suggest the presence of resistant bacteria naturally in pig farms, though it does not take into account the use of antibiotics on these farms prior to the organic raising period.

The use of different types of antibiotics differs according to the infections to be treated and varies according to the age of the pigs [27]. However, knowledge about the effect of the route of administration of antibiotics on the level of resistance is still fragmentary. It is estimated that 90% of antibiotics are given orally, either in food or water [27]. Alali et al. showed a correlation between the amount of tetracycline in pigs’ food and the level of isolates that were resistant to this antibiotic in fecal samples [32], but they found no similar correlation when the antibiotic is given by injection. Another recent study showed that orally administered tetracycline causes the gut microbiota to be more exposed to the antibiotic than by injection, and therefore promotes an increase in antibiotic resistance in the gut [25]. Conversely, the study done by Græsbøll et al. indicated that no significant difference was found in the proportion of resistant coliforms in the feces of pigs between tetracycline administered orally or by injection [33]. The message of these studies, when combined, is that it is necessary to deepen our knowledge on the impact of administration methods for different antibiotic molecules.

### 2.2. Use of Antibiotics in Animal Rearing

#### 2.2.1. Microorganisms to Control in Pigs

Despite the biosecurity measures in place, pathogenic microorganisms may find their way into the rearing section and cause health issues to the herd that require antibiotics. Pathogens can notably cause diseases to the skin, respiratory, digestive, urinary and reproductive systems. Some microorganisms, such as strains of *E. coli, Clostridium perfringens* and *A. pleuropneumonia*, will infect one system while others, like *Streptococcus, Salmonella* and the porcine reproductive and respiratory syndrome virus (PRRSV) will most likely be polysystemic [34].

Disease may be caused by a single organism, but co-infections can also occur with two bacteria or a bacterium and a virus [35,36]. Within the context of animal production, these co-infections make it difficult to identify the culprit. Indeed, biological interactions between microorganisms are likely to influence both the clinical symptoms observed by the producers and the severity of the infection [36]. When an infection occurs, the pathogen is qualified as a primary etiologic agent when it causes the disease. It can be designated as secondary or opportunistic when other microorganisms or conditions first create the conducive circumstances to infect the host [37].

Diseases of the respiratory and digestive systems are particularly problematic in pig production. In 2012, the US Department of Agriculture (USDA) carried out a survey on several pig farms [38]. This survey revealed that respiratory problems were the main cause of mortality in nursery facilities, and in fattening and finishing facilities, where they represent 47.3% and 75.1% of the mortality rate, respectively [38]. Respiratory infections often have multiple factors, which is why the term Porcine Respiratory Disease Complex (PRDC) was introduced to cluster together all the affecting factors. Of these factors, we can state the environment, the genetic background and the infectivity of the culprit microorganisms [39,40]. Many pathogenic agents may be responsible for PRDC; the principal ones were inventoried by Opriessnig et al. [40] and are presented in Table 1. PRDC often implies co-infections between viruses and bacteria or both bacteria and viruses. For example, *Mycoplasma hyopneumoniae* is a primary pathogenic agent known to suppress the host’s immune system, which makes it easier for other pathogens to infect the animal. In pigs, this bacterium may contribute to co-infection with porcine circovirus type 2 (PCV2), the pig influenza virus, *A. pleuropneumoniae*, the herpes viruses, and *P. multocida* [36].

#### 2.2.2. Non-Infectious Factors Influencing Pig Infections

For a pig to get infected, it must come into contact with pathogens. Hygiene management (cleaning) is thus paramount to control the growth and spread of undesirable microbes, which should limit the need for antibiotics. When environmental conditions are not optimal, the animals become stressed, which favours microbial growth and increases the risk of infection [41]. Environmental elements that need to be managed to mitigate stress in pigs include temperature and humidity, ventilation in the barn, animal handling and transport, and changes in the feeding program [42].

Weaning piglets too soon (3 weeks of age) makes the animals more susceptible to infection. Weaning is particularly critical since the piglets go through many changes including being separated from the sow, transport, transition from a liquid to a solid diet, and the presence of new individuals. All those events cause stresses which affect the immune system and makes the animals more prone to infection [43]. Piglets suffer stress during the weaning period, and have only acquired limited immunity at that developmental stage, since maternal immunity acquired at birth dissipates quickly between the third and fourth weeks of life. At that time, piglets have not acquired all their gastrointestinal barriers, including the epithelial barrier, the immune system, and the enteric nervous system, making it easier for bacteria to implement themselves in the digestive system [44]. Weaned piglets are particularly susceptible to post-weaning diarrhea caused by enterotoxigenic *E. coli* (ETEC) [45,46]. As already briefly mentioned elsewhere in the review, many strains of *E. coli* harboring a wide variety of genes and vectors conferring resistance to antibiotics have been isolated from pigs [26,47].

#### 2.2.3. Antibiotic Usage in Pig Production

Four different objectives drive antibiotic usage in animal production. The first is a curative intervention, to treat and heal sick animals [48]. The second is a metaphylactic approach when the antibiotic is given to the entire herd. It aims both to cure sick animals, and to prevent the propagation of the disease to other animals in the herd that are not yet sick. Third is a prophylactic use, when an antibiotic is given to prevent a disease. An example of this is when animals may not present clinical signs, but a particular infection is recurrent over time on a particular production site, so the producers tend to use antibiotics to prevent infection to the new herd [48]. The fourth objective, use of antibiotics as growth promoters, is disappearing. In this case, subtherapeutic doses are notably used to control subclinical infections; which results in better weight gain [49]. Shryock and Page [50] describe the 12 possible modes of action associated with the use of antibiotic as growth promoters. In light of the contribution of this practice towards antibiotic resistance, it is no longer socially acceptable.

The use of antibiotics as a prophylactic measure is widely used in pig production. Of 50 farms located in Belgium, 49 of them use antibiotics, and 93% of the oral or injectable antibiotics were given as prophylactics [48]. It may be difficult to eliminate antibiotics used prophylactically without impairing animal health, particularly at weaning, as already discussed. Diana et al. demonstrated that removing sulfadiazine-trimethoprim (14.4 mg/kg body weight (BW) × day) given prophylactically in the feed of weaned piglets led to an 11.2% increase (significant, *p*  <  0.001) in the use of amoxicillin given curatively (15 mg/kg/BW/day given for 3 days) [51].

#### 2.2.4. Impact of Viral Infections

Even if viral infections cannot be treated with antibiotics, some viruses can cause bacterial co-infections that may require antibiotic treatments [40]. This is the case for the pig influenza virus where the bacterium *S. suis* co-infects pig respiratory tracts with a pneumonia virus. The bacteria or virus alone may cause pneumonia, but the presence of certain virus strains can favour the development of *S. suis* [35]. Hence, to avoid the development of bacterial infections, some producers tend to treat, preventively, all the herd with antibiotics even if the clinical signs are more typical of a viral infection [34].

#### 2.2.5. The Future of Antibiotics in Animal Production

More and more, antibiotics given to farm animals are subject to stringent regulations to counter antibiotic resistance. Many countries, including Canada, Korea, and several countries in the European Union, have completely eliminated the use of antibiotics as growth promoters in animal production [49]. The European Union also made a legislation that will limit the prophylactic and metaphylactic use of antibiotics by 2022 [52]. Even if it is possible to have a more judicious and reduced use of antibiotics, for example by implementing on farm biosecurity measures, it is somewhat difficult to eliminate them completely from animal production because they remain an important solution to treat sick animals. One of the objectives of the One Health approach is to control the development of antibiotic resistance. However, animal health and welfare must remain a priority when disease strikes, and antibiotic treatment may be able to ease the suffering of sick animals. As of now, 16 classes of antibiotics have been identified by the MAPAQ as antibiotics used in pig production. Of those classes, 14 are used in human medicine, two of them (cephalosporins and fluoroquinolones) are considered to be of very high importance [53]. In this context, the province of Quebec has implemented a regulation limiting the use, as a prophylactic measure in animal production, of antibiotics of high importance (Category 1). However, the regulation allows the use of those antibiotics for curative means if no other treatments are available [54].

### 2.3. The Pig Microbiome

It is now known and widely accepted that an organism’s microbiome, which includes living microbiological entities and their by-products [55], plays a crucial role in the general homeostasis of the organism. At the human level, the number of bacterial cells in the microbiota is estimated at 3.8 × 10^13^, while the number of human cells in the body is estimated at 3.0 × 10^13^ [56]. However, it is not just a matter of cell number. The microbiota is involved in several important functions, including the production of short-chain fatty acids (SCFA) [57]. SCFA, resulting from the fermentation of carbohydrates by bacteria in the microbiome, are known to have multiple key functions: major energy contribution [58], regulation of intestinal gluconeogenesis [59], improved antimicrobial activity of macrophages [60] and protection against pathogens by modulating the pH [61]. 

Taking antibiotics alters the composition of the microbiota and reduces the production of SCFA in pigs. A study by Pi et al. showed that a cocktail of antibiotics (ampicillin, gentamicin and metronidazole) significantly decreased the concentration of acetate, propionate, isobutyrate, isovalerate and branched-chain fatty acids in the ileal digesta of growing pigs [62]. Pi et al. demonstrated that taking antibiotics increased the concentration of cadaverine and ammonia, and altered the digestibility of certain amino acids; this suggests a modification in the metabolization of nitrogen and the fermentation of proteins by the intestinal microbiota. The authors also found a change in the microbial composition: a marked decrease for the genera Bifidobacterium, Lactobacillus and Ruminococcus and a significant increase for *E. coli*. This result corroborates the fact that E. coli is a particularly problematic bacterium in terms of antibiotic resistance, as already described.

The study by Che et al. in 2019 investigated the impact of taking a low dose of antibiotic (a premix of chlortetracycline and virginiamycin) on the intestinal microbiota, the production of SCFA and the regulation of genes in colon tissues using a cohort of 28 piglets [63]. A highlight of this study was that antibiotics in low doses had a limited impact on the production of SCFA in the colon, and a negligible impact on the ileum as well. Surprisingly, they demonstrated that using antibiotics caused a shift in the bacterial community potentially involved in the production of SCFA. In the control group, bacteria from the families Prevotellaceae, Spirochaetaceae and Methanobacteriaceae correlated positively with the production of SCFA. However, in the group of pigs fed with antibiotics, the correlation of families Prevotellaceae and Spirochaetaceae disappeared to make way for a complex network involving more bacterial families, and increased the correlation of Methanobacteriaceae with dimethylol butyrate, tetramethyl valerate and caproate.

In fact, Che et al.’s study confirmed Looft et al.’s results, which demonstrated that the microbiota is different according to the sections of the intestine and that antibiotics cause compartment-dependent effects [64]. The microbiological community was found to vary between different sections of the gut. In all sections, the community from the lumen and mucosa was also found to be different. This difference was particularly noticeable in the ileum, where the mucosal community had a much higher diversity, with 299 operational taxonomic units (OTUs) compared to 13 in the lumen [64]. 

According to Looft et al., the response of different taxa to antibiotic treatment also varied in the cecum and colon. The colon had decreased populations of *Treponema*, and increased *Escherichia*, *Lachnobacterium* and *Salsuginibacillus* spp. Those changes were not observed in the ileum. There were differences between the colon and the cecum: while *Turicibacter* was reduced in the colon, Helicobacter was decreased in the cecum, and *Anaeroplasma* and *Paraprevotella* spp. were increased in the cecum. The functions were affected in different ways depending on the gut locations. The dormancy-related genes were significantly reduced in the ileum and the colon, though they were not affected in the cecum [64].

Compartment-dependent effects were observed after a treatment by ileum terminal antibiotic infusion [65]. Indeed, there were some effects specific to the jejunum and the colon. *Bifidobacterium* was decreased in the colon and *E. coli* increased in the jejunum, there was also a decrease in SCFAs in the colon. Zhang et al. also found that the immune response of treated and untreated pigs differed, with treated pigs showing diminished immunoglobulin A, interleukin 8, and interleukin 10 in the colon mucosa. In the jejunum mucosa, immunoglobulin A and G were decreased. This supports the theory that the microbiota plays a crucial role in the shaping of host immunity, and that antibiotic treatment can affect immunity.

It has been hypothesized that a fecal matter transplant (FMT) from a healthy donor could help restore the microbiota after antibiotic treatment [66]. However, antibiotic treatment prior to FMT could undermine the beneficial effect of FMT in piglets [66]. In fact, FMT received after an antibiotic treatment did not restore the immune response or the level of antibiotic resistance in the microbiota to match that of the group that only received FMT.

As mentioned in Section 2.1, the antibiotic administration method was found to influence the impact of treatment on the gut microbiota. Feed-based administration has a greater impact on the ARG level in the gut microbiome and causes a greater shift in microbial population than intramuscular injection [25]. In-feed antibiotics increases the quantity of ARGs in the microbiome. This has been observed for a wide range of antibiotics [67]. Generally, treatment with an antibiotic of a given class tends to lead to an increase of ARGs of this class. Indirect selection was observed to occur for ARGs to antibiotics that had not been administered [4,68]. 

It is important to state that non-treated pigs still have ARGs in their microbiome. Treatment with antibiotics led to an increase of those genes, but they are ubiquitous in the pig gut microbiome and are also present in wild animals [69]. The relative abundance of genes coding for MGEs were observed to increase after antibiotic treatment. Int1, which contributes to the horizontal transfer of ARGs and is often implicated in the carriage of multidrug resistance, is among the elements that are increased by antibiotic treatment [8,70,71].

## 3. From Pig to Environment

Swine farms produce several by-products that can have an impact on the environment, namely pig manure, farm wastewater, and farm air. Manure can be used as fertilizer, and depending on each country’s regulations, wastewater can be released into the environment in various ways. However, it has been observed that pig manure and farm wastewater can contain antibiotic residues, antibiotic resistance genes and antibiotic resistant bacteria [72,73,74]. The quantity of these components is higher when the pigs are treated with antibiotics than when the pigs are not treated. As stated in Section 2.1, this increase is greater when the antibiotics are administered in feed than when they are administered by injection [25].

The dominant ARGs carrying bacteria differ between manure and wastewater. Most multi-drug-resistance genes are carried by *Streptococcaceae* in manure, while the dominant multi-drug resistance carriers in wastewater are part of the *Moraxellaceae* [74]. In both manure and wastewater, *Proteobacteria*, *Firmicutes*, *Actinobacteria* and *Bacteroidetes* were associated with most of the resistance [74]. High temperature composting with thermophilic bacterial agents was shown to reduce both the number of ARGs and integrase genes in pig manure [75], suggesting that it can reduce the ARG spread by manure used as fertilizer for feed crops [75]. Integrase, in particular *int1*, has been correlated with the quantity of many ARGs in pig manure [8,71].

The addition of manure-containing antibiotic residues to soils selects antibiotic-resistant bacteria and increases ARG levels [70]. Manure-amended soils were shown to contain more ARGs than non-amended soils or soils that were fertilized using commercial chemical fertilizer [5]. This could be due to both the introduction of antibiotic-resistant bacteria from the manure, and the selective pressure applied by the residual antibiotics in the manure. This increase is also observed in the crops produced by those soils. Vegetables grown in manure-amended soils were shown to contain ARGs and antibiotic residues [76]. The roots of these vegetables were more contaminated by both antibiotic residues and ARGs than the leaves [76]. There are more ARGs in manure-treated soils, and the quantity of integrase increases in manure-amended soils [70]. This could indicate that manure promotes horizontal transfer of ARGs, which is further supported by an analysis of co-occurrence and co-exclusion of bacteria throughout the samples that showed a higher level of connection between bacteria in manure-amended soils. The clustering coefficient, transitivity and density of the bacterial network increased with higher antibiotic concentrations [77]. These phenomena were reported to last over time. Gao et al.’s study on the effects of organic fertilizers on ARG content in soils, showed that the increase of ARGs in soil was still significant after five years compared to unamended soil [78]. Soils that receive a high volume of manure showed an increase of *sul1* and *tet*(T), which are genes coding for proteins that confer resistance to antibiotics of the sulfonamide and tetracycline classes. This difference, compared to soil receiving mineral fertilizer, was still significant 180 days after application [78]. The possible spread of ARGs to the environment by manure was illustrated by Teng et al.’s study of the contamination of rivers by colistin-resistant *E. coli* in Taiwan. This study showed a correlation between the detection of *E. coli* carrying the *mcr-1* resistance gene in rivers and the density of pigs in the region [79]. 

Many manure treatments have been tested to reduce the amount of ARGs and antibiotics residues in manure and wastewater. Anaerobic digestion has been shown to reduce the tetracycline resistance genes in manure but failed to eliminate it or the tetracycline residues [80]. Ectopic fermentation systems were tested and reduced the abundance of tetracycline resistance genes and metal resistance genes in manure [81]. The use of microbial agents during composting can also reduce the number of ARGs and antibiotic residues [75,82]. A study by Liu et al. reported that a combination of *Phanerochaete chrysosporium*, *Aspergillus niger*, and *Bacillus licheniformis* was effective in removing oxytetracycline and tetracycline residues, reducing them by 87.8% and 93.7%, respectively, and that *P. chrysosporium* was more effective for removing doxycycline and enrofloxacin [75]. A study by Li et al. using a commercial microbial compound during composting found that it could reduce the abundance of ARGs in manure, however, some aminoglycoside and sulfonamide resistance genes were enriched during composting [82]. Several of the enriched genes were correlated with *int1*, which could mean that horizontal transfer was a factor in the enrichment of those genes [82]. This suggests that the microbial compound used has an influence on the type of ARGs that could be removed or enriched [82]. According to current data, one treatment cannot be used to reduce the ARGs of all classes of antibiotics, nor can it be used to completely remove ARGs of a given class. Composting manure before using it as a fertilizer might contribute to reduce the spread of ARGs from the farm to the environment [75,81,82]. However, more studies are required to properly assess the impact of composted manure on soils.

Wastewater treatments achieved similar results [73]. Yang et al. investigated two pig farm water treatment systems: one had an anaerobic tank and a ceramsite biofilter and the other had a plug flow anaerobic reactor, a temporary storage tank, a solid–liquid separation step, an upflow anaerobic sludge blanket, a primary clarifier tank, an anaerobic tank, an aerated tank and a second clarifier tank. While both systems could reduce the abundance of MGEs and ARGs, the concentration in the final effluent was still considerably high after treatment [73]. Zhang et al. demonstrated that treatment of wastewater by nanoscale copper was able to significantly reduce the number of both ARGs and antibiotic-resistant bacteria in wastewater [83]. This treatment was shown to damage bacterial DNA and to last over time, as the level of ARGs was still low four days after treatment [83].

The farm air has an impact on the dispersion of antibiotic residues, ARGs and antibiotic-resistant bacteria to the environment [84]. The presence of antibiotic residues in the air mainly come from particles of in-feed antibiotics and unmetabolized antibiotics released from the manure [85]. Hamscher et al. sampled air dust in a pig-fattening farm over 20 years and demonstrated that the concentration of antibiotics in the air can reach 12.5 mg/kg of dust [85]. They found up to five different types of antibiotic molecules in each sample; the most prevalent were tylosin, sulfamethazine and tetracycline. Bioaerosols transport high amount of bacteria, fungi and viruses. Pilote et al. determined that the countable microbial contamination in the air can reach up to 2 × 10^6^ colony-forming units per cubic metre (CFU/m^3^) and that the contamination mainly comes from the manure [86]. The microbial composition of the bioaerosols tends to vary with the age of the pigs, for example, the abundance of the *Bacteroidetes* and the *Proteobacteria* was higher in the weaning phase than in the finishing and farrowing phase [87]. Pig farms need good ventilation for good quality air to prevent the transfer of particles that increase infection and that can lead to antibiotic resistance to the environment in the end after treatment. Zhang et al. revealed that the antibiotic-resistant bacteria from the air were still found at 150 m altitude downwind of the farm [88]. It is also known that external manure storage produced bioaerosols that facilitate the transfer of ARGs to the environment [84].

## 4. From Pig to Humans

After enrichment in the pig microbiome, ARGs can be transmitted to humans. Pig farms and slaughterhouse workers have a different microbiome than the rest of the population, and even than broiler chicken farmers [89]. Their fecal resistome contains significantly more tetracycline, β-lactam and macrolide resistance genes (*p* < 0.05). However, their fecal resistome differs from the pig fecal resistome, suggesting either that direct contamination from the feces is not the main factor of transmission, or that the microbiome of humans reacts differently from that of pigs. A higher amount of time spent on the farms correlated with higher ARG content in the fecal resistome.

Similar changes were observed in the human gut microbiome after a month on a farm [90]. Those changes seem not to last over time, as the microbiome reverts to its original state in a matter of months after departure from the farm. The changes associated with a visit to a swine farm correlated with the species and genes present in the swine farm environment.

Among the most worrisome ARG transmission events yet documented is the transmission of colistin resistance genes *mcr-1* [91]. *E. coli* carrying this gene was isolated from both pigs and farmers, and they were highly similar, suggesting a common origin. As colistin is an antibiotic of high importance in treatment of human infections, the acquisition of *mcr-1* by pathogens could lead to serious public health issues. Indeed, the development of resistance to clinically used antibiotics could decrease the efficiency of the treatment of various human diseases caused by bacteria and increase the mortality rates associated with these diseases [79,92,93,94].

In addition to the gut microbiota, the nasopharyngeal microbiota of pig farmers is affected by exposure to pigs [95], and was shown to be more similar to the microbiota of the farm air than to the nasopharyngeal microbiota of humans that were not exposed to pig farms. Pig farmers’ microbiota also had more antibiotic- and zinc-resistance genes than the control group.

Humans can be exposed to ARGs both by direct contact with pigs and by consuming vegetables that were cultivated on soils that have been fertilized with pig manure and swine farm wastewater. As already mentioned, these vegetables contain more ARGs than vegetables produced on soils that were fertilized by commercial chemical fertilizers [76]. Moreover, soils fertilized with manure contained resistant strains of *Rickettsiales*, which is a human pathogen. Therefore, it is possible that humans might be contaminated by vegetables grown on such soils [76].

Randad et al.’s study on the evolution of *Staphylococcus aureus* (*S. aureus*), which infects pigs and humans, showed that the strains of resistant *S. aureus* in pigs and humans are closely related [94]. This suggests that there could be a transmission of antibiotic-resistant pathogens from pigs to humans. Randad et al. also observed that most strains of *S. aureus* isolated in North Carolina were resistant to multiple antibiotics.

## 5. From Pig to Meat

Once the animal has reached the target slaughter weight, pre-slaughter management procedures are set into place. Shipping records, including those related to safety and to the preventive control plan based on Hazard Analysis and Critical Control Points (HACCP), are prepared. These procedures include, for example, feed withdrawal to avoid transport-related sickness and viscera perforation at evisceration, grouping animals in advance to reset hierarchical position among the newly formed group to be transported [96], and verification that antibiotic withdrawal time is met for the animals treated with antibiotics [97,98,99,100]. This antibiotic withdrawal time is important to avoid antibiotic residues in the meat; surveillance programs to that effect are in place in many countries, including Canada, to make sure this requirement is met [101]. When properly followed and monitored, antibiotic residues can be effectively mitigated as a chemical safety risk [100]. Nevertheless, Roudaut et al.’s study in France revealed that less than 0.3% of the pig carcasses tested contained antibiotic residues above the maximum residue limit [102]. Similar surveillance data were reported in Quebec in 2011 to 2016. In meat and poultry, only 8 out of 1113 samples were positive for the detection of veterinary drugs, and none were above the maximum residue limit except for one sample from boar [103].

The edible tissues of healthy animals are considered to be sterile or to contain very limited numbers of microorganisms, except for lymph nodes, due to their immune functions in the body [104]. Maintaining good herd health status and management are paramount to secure the safety of meat supply. During carcass dressing, it is the surface of the carcass and the retail cuts that get contaminated from organisms coming from the hide/skin of the animal, gut content, workers’ hands and the slaughter environment [96]. It is then of no surprise that if animals raised for meat consumption harbour antibiotic-resistant microorganisms that those will find their ways into the meat to be consumed. Isolation of antibiotic-resistant microorganisms from various types of meat has been abundantly reported in the literature [105,106,107,108,109], including for pork [110,111].

The use of antibiotics, whatever the purpose, will create a selective pressure for resistant bacteria; antibiotic resistance was observed in pathogens [112] and spoilage [113] as well as beneficial microorganisms found on meat, such as lactic acid bacteria [114]. 

Judicious use of antibiotics reduces the selective pressure, thereby decreasing the incidence of antibiotic-resistant bacteria, as was observed by Sapkota et al. [115] when conventional poultry farms transitioned to organic practices. To that effect, they even suggest that the incidence of antibiotic-resistant bacteria in livestock could be reversible with the reduction of antibiotic usage. Haskell et al. [116] studied the incidence of antibiotic-resistant *S. aureus* in conventional and antibiotic free chicken and turkey meat and reported a lower incidence in antibiotic-free poultry meat. However, even a limited presence of a resistant and persistent subpopulation can resurface upon antibiotic usage for therapeutic purposes, or else, since it remains more adapted to the selective pressure [101].

Since antibiotic resistance is carried by wild antibiotic-producing organisms, zero antibiotic resistance transfer seems impossible, even if no more antibiotics were to be used by humans. To that effect, Chen et al. [117] compared the number of ARGs in wild boar gut microbiome with Duroc pigs. They found a significantly lower number of ARGs in eight wild boars captured in the mountains of the Jiangxi Province. Similarly, Guerrero-Ramos et al. [118] found ARGs in enterococci isolated from wild roe deer, boar, rabbit, pheasant, and pigeon hunted in North-Western Spain. The occurrence of antibiotic resistance in wild animals suggests that either it may exist naturally in remote environments, or that those environments may have been contaminated by human (likely agricultural) activities, or a combination of both; the contribution of each factor remains difficult to assess and separate. In another study [119], 16 of the 229 *E. coli* isolates recovered from frozen cuts of red deer, roe deer and wild boar meat sampled in a game handling establishment in Germany demonstrated resistance to at least one of the antimicrobial agents tested. Despite a lower prevalence of *E. coli* and of antibiotic-resistant *E. coli* in game meat compared to conventional meat, part of the resistant strains recovered may originate from the processing environment. Despite existing cleaning programs, microorganisms resistant to antibiotics have been recovered from equipment in slaughter and meat processing facilities [111,120]. As sampling goes down the processing line, detection of bacteria bearing resistance genes decline [120]. Part of the persistence in the meat plant environment may, at least in part, be explained by cross-resistance with sanitation products, particularly through multidrug efflux pumps [101,110,121]. In an early (1988) study, Mattila et al. [122] investigated the transfer of antibiotic resistance in *E. coli* on the surface of meat at 20 and 4 °C. They concluded that the lack of transfer between donor and recipient cells was due to the sessile growth on the meat surface which prevented the cell contact necessary for the transfer of genetic materials. Van Meervenne et al. [123] indicated that a threshold cell density is necessary for the transfer of plasmid-bearing antibiotic resistance on cooked ham. They observed plasmid transfer from *Lactobacillus sakei* subsp. *sakei* to *Listeria monocytogenes* on cooked ham at abused refrigeration temperatures (7 and 10 °C), but Walsh et al. [124] did not observe ampicillin resistance transfer from *Salmonella* Typhimurium DT104 to *E. coli* K12 in Luria Bertani broth, milk and ground meat at a proper refrigeration temperature of 4 °C with a high inoculation level of 10^5^ cfu/ml or g. With respect to modified atmosphere packaging, transconjugants were observed at low cell density under 100% N_2_, but not with air and 50% CO_2_-50% N_2_ after 10 days of incubation at 7 °C [123].

Amador et al. [125] found *Enterobacteriaceae* with antibiotic resistance in deli meats which were either smoked and/or salted or boiled. Of the 19 samples they collected, only two were prepackaged. Antibiotic resistance varied from negative to 1 × 10^8^ cfu/g and the smoked meat were less susceptible to harbour antibiotic-resistant organisms. Similarly, Fijalkowski et al. [126] isolated antibiotic-resistant staphylococci from ready-to-eat meat products, including pork ham, chicken cold cuts, pork sausage, salami, pork luncheon meat, sliced upon request in five randomly selected butcher shops. This means that the luncheon meats collected were not pre-packaged and part of the contamination may come from the shop environment. Alban et al. [112] studied the prevalence of the multi-resistant *Salmonella* Thyphimurium DT104 in dry-cured pork sausages which are not typically cooked, but fermented. Their risk assessment analysis suggests that the product will seldom harbour the organism provided it is present in low numbers in the raw meat (≤50 per 400 cm^2^ surface), and that a 2–3 log-unit reduction is to be expected during processing.

In surveillance programs by the Canadian Integrated Program for Antimicrobial Resistance Surveillance (CIPARS), *E. coli* that are resistant to at least one antibiotic in pigs and pork meat at the farms, abattoirs, and retail were monitored (Table 2). From the data collected by CIPARS from 2002 to 2017, antibiotic resistance in *E. coli* declined over time at the farm and at the abattoir. Data at the abattoir were similar to the data collected at the farms until 2011; thereafter it was 1 to 7% higher at the abattoirs. Data collected on pork chops sampled from retail markets were lower than those obtained at the farms and at the abattoirs, which suggests that as the collection site is away from where the antibiotic is used, the contaminating microorganisms from the processing line are less antibiotic resistant. The prevalence of *E. coli* that is resistant to at least one antibiotic was half to one third compared to *E. coli* observed at farms and abattoirs until 2009; thereafter, it was half or a little more than half as prevalent. These data suggest that the prevalence decreased as the industry responded actively to a more judicious use of antibiotics in animal rearing, but contamination from the farms has established itself along the food chain. 

Similarly, Rahimi et al.’s study conducted in the Isfahan and Shahrekord provinces of Iran on antibiotic resistance in *Shigella* in meat and meat products reveals a similar pattern [127]. Incidence of antibiotic resistance is higher in raw meat than in processed meat. Nevertheless, McMahon et al. [128] demonstrated that sublethal stress processes, commonly used in food preservation, do alter antibiotic resistance. Some, such as high temperature (45 °C), decrease antibiotic resistance, whereas others increase antibiotic resistance, even after removal of the stress, which suggests that the bacteria have developed an increased resistance that lasts over time. With *Cronobacter sakazakii*, cells stressed by desiccation (40 °C for 2 h and held at 21 °C for 4 d) were more sensitive to streptomycin, gentamicin, kanamycin, tetracycline, vancomycin, ampicillin, enrofloxacin and doxycycline, but less sensitive to neomycin and amoxicillin. For cold (4 °C for 24 h)-stressed cells, increased sensitivity to 8 out of the 13 antibiotics tested was observed. Heat stress (55 °C for 5 min) caused the greatest sensitivity increases of the five stress-inducing methods tested. Sensitivity was increased to all 13 antibiotics tested except for two out of the five strains tested for streptomycin and three for neomycin [129]. The heat stress applied by these conditions were well below the cooking temperature of processed meat, which is typically a core temperature of 71 °C.

## 6. Alternatives to Antibiotics

Reduction in antibiotic usage leads to a reduction in the number of resistant strains. However, for animal welfare reasons, pork producers and veterinarians believe that these molecules remain essential [130]. Therefore, it is necessary to develop new strategies to reduce our dependence on antibiotics. 

Although some bacteria are pathogenic and can cause diseases, others can have beneficial effects on health. Using bacteria, including those that produce lactic acid, as probiotic agents is a strategy that is well advanced and for which several recent studies demonstrate a high efficiency in pigs [131,132,133,134]. A similar approach is to use bioactive molecules produced by probiotic bacteria (proteobiotics). For example, Nordeste et al. demonstrated that the addition of lyophilized culture supernatant of the probiotic strain *Lactobacillus acidophilus* La-5 to piglet feed reduced the risks of collibacilosis [135].

Using bacterial viruses, namely bacteriophages, is also a promising alternative which has been increasingly tried on pigs and seems to work well to reduce the load of pathogens and to increase growth [136,137,138]. For example, Zeng et al. have shown that adding a cocktail of phages globally targeting *Salmonella*, *E. coli*, *C. perfringens* and *S. aureus* to pig feed significantly increased the average daily gain and feed intake and decreased the feed/gain ratio and the risks of diarrhea in weanling piglets [137].

Several metals, such as zinc and copper have important antimicrobial effects [139], and as discussed elsewhere [140], can be used as alternatives to antibiotics in farm animals. The use of high doses of zinc oxide (ZnO) is particularly effective in reducing the risk of post-weaning diarrhea in piglets [141]. However, there is increasing evidence that the addition of metals, including ZnO, at high doses in animal feed, causes selection of antibiotic resistance genes through cross-resistance, and promotes the transfer of these resistance genes between bacteria [140,142,143,144]. Research is being done to try to find more eco-responsible forms of zinc to retain the benefit of ZnO. For example, Oh et al. demonstrated that 200 ppm of ZnO nanoparticles have the same effect as 2500 ppm of ZnO [145].

As discussed in several other literature reviews and articles [146,147,148,149,150,151,152,153,154], numerous other alternatives to antibiotics have also been explored, such as the addition of proteins, fibers, amino acids, enzymes, organic acids, essential oils, antimicrobial peptides, egg yolk antibodies, and spray-dried plasma to animal feed. Finally, it is interesting to note that several of these alternatives have also been explored for humans [146,147] and that research in animals and humans is mutually supportive.

## 7. Conclusions and Perspectives

This review indicates that the use of antibiotics has been shown to be effective to treat bacterial infections for decades, both in a curative and prophylactic context. Antibiotics can also influence the zootechnical performance of animals, such as growth. The problem does not lie with antibiotic residues in the meat, but with antibiotic resistance in many spheres of activity, including pig farming. Resistance to antibiotics is a natural phenomenon independent of human actions. In his opening keynote remark to the 2000 ASM meeting, Julian Davis highlighted that the antibiotic producer strains must have immunity genes, otherwise it is suicide. Therefore, resistance genes occur naturally in the environment and have the potential to be transferred which makes zero antibiotic resistance utopic. Our extensive use of these molecules favoured a selection for resistant strains and led to a considerable increase in the number of these strains. On a hopeful note, some studies suggest that this phenomenon could be somewhat reversible, or at least attenuated, and therefore it may be possible to return to near basal level even though resistant strains may remain in low numbers within the population with the potential to resurface upon selective pressure. In any case, it is crucial to maintain and improve biosecurity measures: these are still our first line of defense to protect herds against infectious diseases and help to limit the need for antibiotics. 

## Figures and Tables

**Table 1:** Main pathogenic agents responsible for Porcine Respiratory Disease Complex.

	Pathogenic Agent
*Bacteria*	*Actinobacillus pleuropneumoniae*
	*Actinobacillus suis*
	*Bordetella bronchiseptica*
	*Mycoplasma hyopneumoniae*
	*Pasteurella multocida*
	*Salmonella spp.*
	*Streptococcus suis*
*Virus*	*Swine influenza*
	*Porcine reproductive and respiratory syndrome (PRRS)*
	*Herpesvirus/Pseudorabies*
	*Porcine circovirus type 2 (PCV2)*

**Table 2:** Prevalence of *E. coli* that are resistant to one or more antibiotics collected according to the surveillance program of the Canadian Integrated Program for Antimicrobial Resistance Surveillance (CIPARS) for pigs and pork meat ^a^.

Year	Farm		Abattoir		Retail	
	%	NB^b^	%	NB	%	NB
2002			97	38/39		
2003			98	153/155	50	151/301
2004			99	142/143	54	321/593
2005			99	163/164	40	316/797
2006	99	459/462	98	115/117	38	288/763
2007	100	612/612	98	93/95	36	310/871
2008	99	481/486	100	150/150	32	317/979
2009	99	695/701	98	160/263	29	323/1097
2010	84	1402/1673	83	165/199	47	118/250
2011	83	1389/1667	85	161/190	54	231/431
2012	86	1333/1553	89	164/184	50	97/193
2013	83	1313/1573	84	143/171	41	91/221
2014	81	1351/1672	88	141/161	54	176/323
2015	77	385/500	79	152/192	57	102/179
2016	79	428/544	84	152/182	44	62/140
2017	77	374/484	80	132/164	44	51/115

^a^ Samples at the farm were from feces, at the abattoir from caecum material, and at the retail outlet, from chops. ^b^ NB = number of positive over the total number of samples collected.
